# Development of Sensitive R6G/AuNCs Ratiometric Fluorescent Probes for the Detection of Biogenic Amines in Fish Products

**DOI:** 10.3390/ijms26010139

**Published:** 2024-12-27

**Authors:** Yutong Huang, Simiao Zhang, Mei Zhou, Xiaokang Xu, Weiqing Sun, Jing Ma, Long Wu

**Affiliations:** 1 College of Life Science, Yangtze University, Jingzhou 434023, China; 2 School of Food Science and Engineering, Key Laboratory of Tropical and Vegetables Quality and Safety for State Market Regulation, Hainan University, Haikou 570228, China

**Keywords:** ratiometric fluorescent probes, rapid detection, biogenic amines, aquatic products, food safety

## Abstract

Biogenic amines (BAs), produced in fish and seafood due to microbial contamination, pose significant health risks. This study introduces a novel ratiometric fluorescent probe, synthesized by integrating rhodamine 6G(R6G) and gold nanoparticles (AuNCs), for the sensitive and specific detection of BAs. The probe operates on the principle of BAs hydrolysis, catalyzed by diamine oxidase, to produce hydrogen peroxide (H_2_O_2_), which selectively quenches the fluorescence of AuNCs at 620 nm, while the fluorescence of R6Gat 533 nm remains unaffected. The ratio of I_620_/I_553_ demonstrated excellent linearity with a wide dynamic range (1–1000 μM) and a low detection limit of 0.1 μM. Validation using grass carp samples showed high recovery rates (97.57% to 104.29%), confirming the probe’s efficacy and potential for practical application in food safety monitoring.

## 1. Introduction

Biogenic amines (BAs), a group of biologically significant alkaline nitrogenous compounds, play pivotal roles in various physiological processes, including protein and hormone synthesis, DNA replication, modulation of allergic responses, and hematopoiesis, thereby maintaining cellular homeostasis [1,2]. The human gastrointestinal tract is equipped with monoamine oxidases, diamine oxidases, and polyamine oxidases that metabolize ingested BAs. An imbalance in the metabolic capacity of these enzymes, either due to saturation or inhibition, can precipitate cases of food poisoning [3]. The conditions prevalent during the fishing, transport, and marketing of fish—rich in microorganisms—foster an environment conducive to the proliferation of decarboxylase enzymes, leading to the decarboxylation of amino acids and subsequent BA formation [4,5]. Consequently, BAs are widely recognized as biomarkers indicative of microbial spoilage [6]. In response, numerous countries, regions, and regulatory bodies have established stringent limits on BAs in seafood products.

Conventional analytical techniques for quantifying BAs encompass high-performance liquid chromatography (HPLC) [7], capillary electrophoresis (CE) [8], mass spectrometry (MS) [9], and gas chromatography (GC) [10]. These methodologies, while accurate, are often hindered by the need for sophisticated instrumentation, laborious sample preparation, and specialized technical expertise, impeding their utility for rapid, on-site analysis of aquatic products. To address these limitations, alternative approaches such as enzyme-linked immunosorbent assays (ELISA) [11], electrochemical biosensors [12,13,14], nucleic acid aptamer-based assays [15], and molecularly imprinted polymer-based assays [16] have also been developed. These newer methods, while offering simplified operation and enhanced portability, still fall short in terms of sensitivity and stability.

Fluorescence-based detection offers a compelling alternative, combining high sensitivity, excellent stability, cost-effectiveness, and rapid analysis. Ratiometric fluorescent probes, emitting at two distinct wavelengths, leverage an internal calibration mechanism that minimizes the impact of external factors such as environmental conditions, instrumental variations, and probe concentration. This inherent self-referenced design endows ratiometric probes with superior anti-interference capabilities compared to their single-signal counterparts [17]. Gold nanoparticles (AuNCs), with their robust photostability and facile synthesis, serve as an ideal fluorescent component. Bovine serum albumin (BSA), rich in functional groups like -COOH, -NH_2_, and -SH, not only stabilizes AuNCs but also enhances their fluorescence and biocompatibility, making it an optimal protective and reducing agent [18]. The ease of functionalization of BSA-stabilized AuNCs further broadens their applicability in nanobiosensing [19,20].

**Scheme 1:**
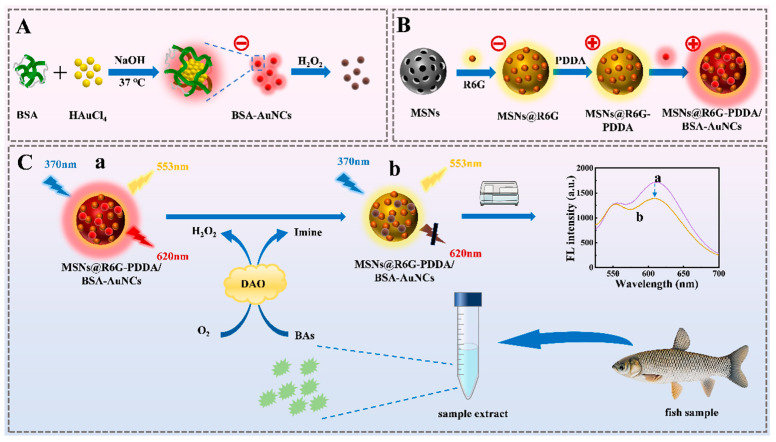
(**A**) Preparation of BSA-AuNCs; (**B**) preparation of fluorescence probe based on MSNs@R6G-PPDA/BSA-AuNCs; and (**C**) principle for detection of BAs.

In this study, we introduce a novel ratiometric fluorescent probe, synthesized by integrating BSA-AuNCs with MSNs@R6G-PDDA, for the detection of BAs. MSNs possess excellent loading capacity due to their porous structure, which provides a high Brunauer–Emmett–Teller (BET) surface area, enabling the adsorption of more R6G molecules and thereby enhancing the fluorescence effect. PDDA, a positively charged polyelectrolyte, imparts a positive charge to the surface of MSNs@R6G, facilitating the subsequent binding with negatively charged BSA-AuNCs. The probe’s detection mechanism is depicted in Scheme 1. The red-emitting BSA-AuNCs and the yellow-emitting MSNs@R6G-PDDA exhibit a maximum fluorescence at 620 nm and 553 nm, respectively, operating independently without spectral interference. When biogenic amines (BAs) are hydrolyzed by diamine oxidase (DAO), the resulting H_2_O_2_ induces the oxidation of gold nanoclusters (AuNCs), causing their fluorescence to burst (Scheme 1C(b)). In contrast, the fluorescence of R6G remains unaffected (Scheme 1C(a)). This approach, by adopting the fluorescence intensity ratio (I_620_/I_553_) as a function of biogenic amine concentration, enables the sensitive and accurate detection of BAs with high efficiency and precision.

## 2. Results and Discussion

### 2.1. Characterization of Ratiometric Fluorescent Probe

TEM was used to observe the microstructure as well as morphology of the synthesized materials. As in Figure S1, the MSNs were spherical with an ordered mesoporous structure.

**Figure 1:**
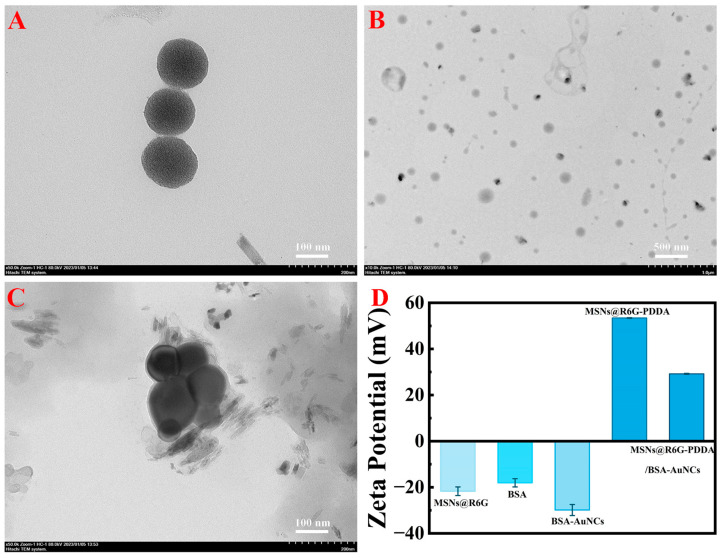
TEM images of (**A**) MSNs@R6G; (**B**) BSA-AuNCs; and (**C**) MSNs@R6G-PDDA/BSA-AuNCs; and (**D**) zeta potential of materials.

As shown in Figure 1A, MSNs@R6G was in the form of smooth and regular spherical shape with uniform particle size, and there was no significant difference in morphology and particle size compared with MSNs. This indicates that the introduction of R6G does not alter the physical properties of MSNs. As shown in Figure 1B, the BSA-AuNCs are uniformly dispersed and present a nearly spherical structure, and the morphology is consistent with previous descriptions in the literature [21]. It can also be seen (in Figure 1C) that the dispersion of the bound MSNs@R6G-PDDA/BSA-AuNCs deteriorated with irregular and rough surfaces and larger sizes. This indicates the successful adsorption between BSA-AuNCs and MSNs@R6G-PDDA by electrostatic interaction; i.e., the ratiometric fluorescent probe was successfully synthesized.

The zeta potential was then measured to illustrate this and the results are shown in Figure 1D. Both MSNs and R6G are negatively charged, which gives a potential of −21.7 mV for MSNs@R6G. When the cationic polymer PDDA was added, the zeta potential became +53.4 mV. The zeta potentials of BSA and BSA-AuNCs were −18.1 mV and −29.9 mV, respectively. When MSNs@R6G-PDDA binds to BSA-AuNCs because of electrostatic interactions, the zeta potential becomes +29.2 mV. This indicates that ratiometric fluorescent probes were successfully synthesized.

**Figure 2:**
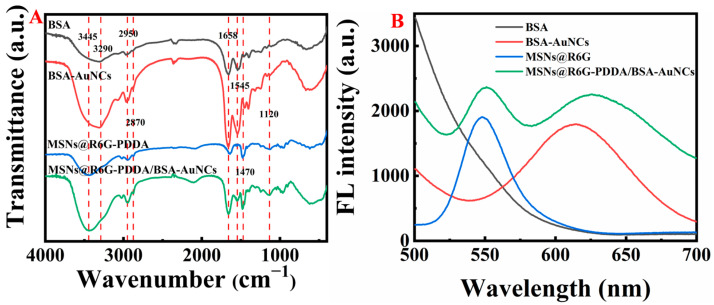
(**A**) FT-IR spectra; and (**B**) fluorescence emission spectra of materials.

The structure of the substance was characterized using FTIR spectroscopy and the results are shown in Figure 2A. In the IR spectra of BSA-AuCNs, there are characteristic absorption peaks at 3290 cm^−1^, 2950 cm^−1^, 1658 cm^−1^, and 1545 cm^−1^ originating from the bending and stretching vibrations of -OH, N-H, C=O, and Au-N, respectively [22]. The new characteristic peaks observed in MSNs@R6G-PDDA compared to MSNs@R6G at 2870 cm^−1^, 1470 cm^−1^, and 1120 cm^−1^ correspond to the C-H stretching vibration in CH_3_, the asymmetric deformation of O-H, and the C-N stretching vibration, respectively [23]. This indicates that MSNs@R6G was successfully modified by PDDA functionalization. The presence of characteristic absorption peaks of MSNs@R6G-PDDA and BSA-AuNCs in MSNs@R6G-PDDA/BSA-AuNCs, as well as the broadening of the absorption peaks at 3700 cm^−1^~3100 cm^−1^, demonstrated the successful modification of BSA-AuNCs on the surface of MSNs@R6G-PDDA.

The fluorescent properties of the material were analyzed. As shown in Figure 2B, BSA has no emission peak in the 500 nm~700 nm emission band, BSA-AuCNs has an emission peak at 620 nm, and MSNs@R6G has an emission peak at 553 nm. MSNs@R6G is modified by PDDA functionalization and binds to BSA-AuNCs through electrostatic interaction. There are two emission peaks at 553 nm and 620 nm, respectively. Moreover, the two peaks are far apart and do not affect each other, indicating that the ratiometric fluorescent probe was successfully synthesized. In addition, the excitation–emission matrix shows that the MSNs@R6G has a maximum fluorescence intensity at around 553 nm (Figure S2A), and the top right inset shows MSNs@R6G fluorescing yellow under UV light. As shown in Figure S2B, BSA-AuNCs fluoresce in red at around 620 nm by the maximum fluorescence intensity, as shown in the upper right inset. As shown in Figure S2C, MSNs@R6G-PPDA/BSA-AuNCs have the maximum emission fluorescence intensities at both 553 nm and 620 nm, and the inset shows that the composite material fluoresces red under UV light.

### 2.2. Feasibility Analysis of Ratiometric Fluorescent Probe Detection of H_2_O_2_

**Figure 3:**
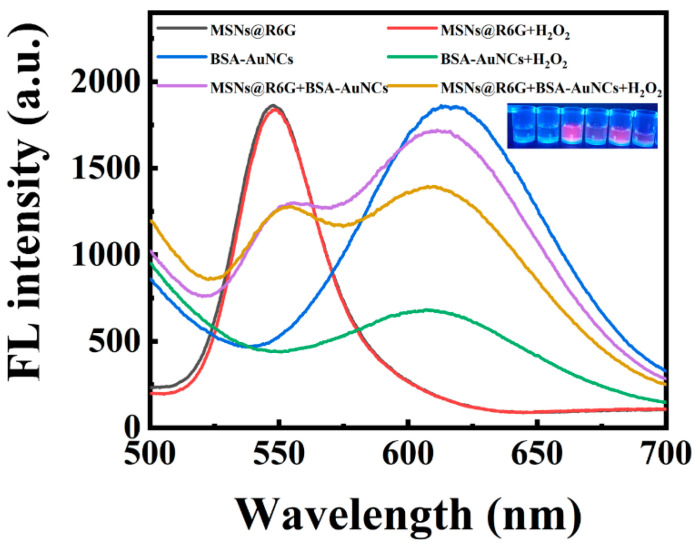
Fluorescence emission spectra of MSNs@R6G; MSNs@R6G + H_2_O_2_; BSA-AuNCs; BSA-AuNCs + H_2_O_2_; MSNs@R6G + BSA-AuNCs; and MSNs@R6G + BSA-AuNCs + H_2_O_2_ (inset: the corresponding photograph of BSA-AuNCs under 365 nm UV light).

In order to verify the feasibility of MSNs@R6G-PDDA/BSA-AuNCs for the detection of H_2_O_2_, a control experiment was designed, and the results are shown in Figure 3. Although MSNs@R6G fluoresce only a lighter yellow under UV lamp, the MSNs@R6G has a strong fluorescence signal at 553 nm due to the high quantum yield of the MSNs@R6G. The fluorescence intensity of MSNs@R6G was not significantly altered by the addition of H_2_O_2_, and there was no significant difference in the fluorescence intensity of MSNs@R6G under UV lamp irradiation compared with that of MSNs@R6G without the addition of H_2_O_2_. This indicates that H_2_O_2_ does not affect the stability of the MSNs@R6G and causes it to be fluorescently quenched. Although BSA-AuNCs have strong red fluorescence under ultraviolet lamp irradiation, the quantum yield is not high. Therefore, the fluorescence intensity of BSA-AuCNs at 625 nm is about the same as that of MSNs@R6G. For MSNs@R6G, the fluorescence intensity did not change greatly after mixing with BSA-AuCNs, but the maximum emission peak of BSA-AuNCs was blue-shifted. This may be due to the dissociation of some of the gold atoms caused by the conformation of BSA [22]. And it still shows red fluorescence under UV lamp irradiation. The fluorescence intensity of BSA-AuNCs decreased at 620 nm after the addition of H_2_O_2_, but there was no significant decrease in the fluorescence intensity of MSNs@R6G at 553 nm. Red fluorescence was observed to be quenched under the irradiation of an ultraviolet lamp. The above experiments show that H_2_O_2_ only quenches the fluorescence of BSA-AuNCs, so this ratio fluorescent probe can be used for the detection of H_2_O_2_. DAO can catalyze the production of H_2_O_2_ from BAs, so it is feasible for ratiometric fluorescent probes to detect BAs indirectly by enzyme cascade.

### 2.3. Optimization of Conditions for the Preparation of Ratiometric Fluorescent Probes

To enable the ratiometric fluorescent probe to detect BAs for an optimal fluorescence response, the dilution of BSA-AuNCs, the addition ratio of MSNs@R6G to BSA-AuNCs, and the incubation time and incubation temperature between MSNs@R6G-PPDA and BSA-AuNCs were optimized.

**Figure 4:**
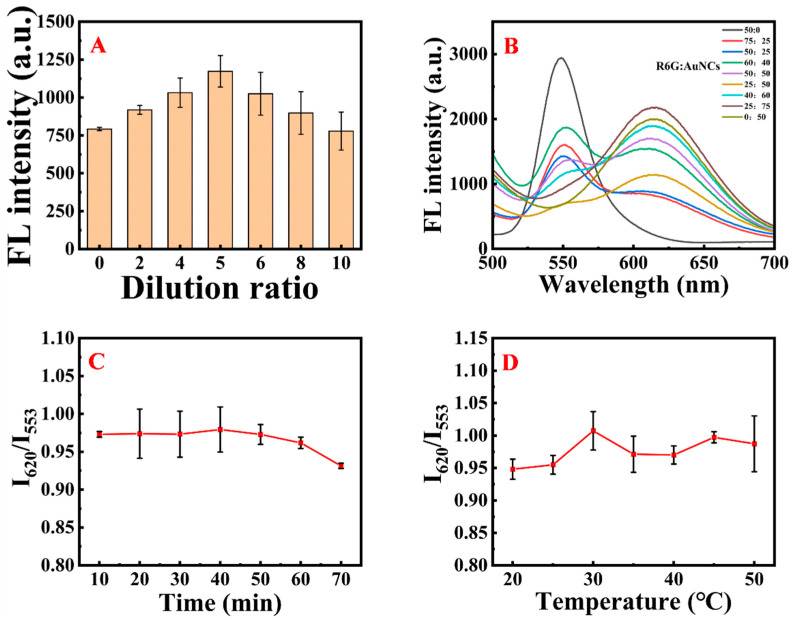
(**A**) Fluorescence intensity measured after 0 (25.3 mg/mL), 2 (12.65 mg/mL), 4 (6.33 mg/mL), 5 (5.06 mg/mL), 6 (4.22 mg/mL), 8 (3.16 mg/mL), and 10-fold (2.53 mg/mL) dilution of BSA-AuNCs stock solution (inset: the photograph of BSA-AuNCs under natural light and 365 nm UV light); (**B**) fluorescence emission spectra of different addition ratios between MSNs@R6G and BSA-AuNCs; (**C**) optimization of incubation time; and (**D**) optimization of incubation temperature.

The BSA-AuNCs were diluted 0, 2, 4, 5, 6, 8, and 10 times, respectively, and the fluorescence intensity was detected using a fluorescence spectrophotometer at an excitation wavelength of 370 nm. As shown in Figure 4A, the fluorescence intensity of BSA-AuNCs initially increased and then gradually decreased with increasing dilution. This is because the fluorescence intensity of AuNCs is concentration-dependent: it increases within a certain range but decreases when the concentration becomes too high, causing a self-quenching effect. A five-fold dilution (5.06 mg/mL) of BSA-AuNCs was ultimately used for subsequent experiments.

Ratiometric fluorescent probes are extremely dependent on the fluorescence intensity ratio between two different fluorescent signals. Therefore, the addition ratio of MSNs@R6G and BSA-AuNCs is particularly important when synthesizing ratiometric fluorescent probes. As shown in Figure 4B, MSNs@R6G and BSA-AuNCs have single emission fluorescence at 553 nm and 625 nm, respectively. After mixing, emission peaks appear at both 553 nm and 620 nm. And the fluorescence emission peak at 553 nm was more intense when the MSNs@R6G was added in too high a proportion, and the fluorescence intensity around 620 nm was greater when BSA-AuNCs was added in too high a proportion compared with free BSA-AuNCs, the emission peaks of BSA-AuNCs appeared blue-shifted after mixing. This indicates that the conformational changes of BSA occurred during the mixing process, leading to the dissociation of some gold atoms, which changed the photophysical properties of BAS-AuNCs and led to the blue shift. During the detection of BAs, the fluorescence of BSA-AuNCs was burst with an increasing BA concentration, and the fluorescence of MSNs@R6G was not affected. Therefore, it is necessary to select the addition ratio when the fluorescence intensity of BSA-AuNCs is slightly greater than that of MSNs@R6G, and the optimal addition ratio of MSNs@R6G to BSA-AuNCs was finally determined to be 50:50.

As shown in Figure 4C, I_620_/I_553_ gradually increased as the incubation time of MSNs@R6G-PPDA with BSA-AuNCs increased. This suggests that more of MSNs@R6G-PPDA bind to BSA-AuNCs with increasing time, with a maximum value of I_620_/I_553_ when the incubation time reaches 20 min. Subsequently, I_620_/I_553_ began to decrease with increasing incubation time, which may be due to the fact that too long an incubation time would result in BSA-AuNCs escaping from MSNs@R6G-PDDA. Finally, 20 min was chosen as the incubation time for MSNs@R6G-PPDA with BSA-AuNCs.

The incubation temperature also had an important effect on the adsorption between MSNs@R6G-PDDA and BSA-AuNCs. As shown in Figure 4D, I_620_/I_553_ increases as the temperature rises. When the temperature reached 30 °C, I_620_/I_553_ was the maximum value. When the temperature continued to increase, I_620_/I_553_ instead decreased, which may be due to the fact that higher temperatures lead to a change in the conformation of the BSA and, thus, to the cleavage of the gold atoms. Thus, 30 °C was chosen as the incubation temperature for MSNs@R6G-PPDA with BSA-AuNCs.

By optimizing the above experimental conditions, MSNs@R6G-PDDA/BSA-AuNCs ratio-based fluorescent probes were synthesized for subsequent experiments.

### 2.4. Stability Evaluation of Ratiometric Fluorescent Probes

Stability is an important indicator of whether a ratiometric fluorescent probe can be used for real-world detection. In this work, the effects of photostability, storage stability, thermal stability, and pH on the stability of the probe were investigated.

Long-term exposure to light of fluorochromes will cause irreversible changes in the photochemical properties, and the fluorescence signal may be sharply reduced, so ratiometric fluorescent probes need to have good photostability. In order to explore the photostability of ratiometric fluorescent probes, I_620_/I_553_ was obtained by fluorescence determination by irradiating MSNs@R6G-PDDA/BSA-AuNCs under a 365 nm UV lamp of a camera obscura analyzer for 0, 5, 10, 20, 40, and 60 min, respectively. As shown in Figure S3A, I_620_/I_553_ did not change significantly due to the irradiation of the ultraviolet lamp, indicating that the photostability of the fluorescent probe with this ratio was good.

In order to explore the storage stability, MSNs@R6G-PDDA/BSA-AuNCs were stored at 4 °C, and I_620_/I_553_ was obtained by fluorescence assay on days 1, 4, 10, and 40. As shown in Figure S3B, the probe did not change significantly in I_620_/I_553_ at 1~10 days. After 40 days of storage, I_620_/I_553_ decreased significantly, indicating fluorescence quenching. This is due to the presence of dissolved oxygen in the solution, which reacts with the groups on the surface of BSA-AuNCs, which changes the charge and structure of the surface of BSA-AuNCs, and eventually leads to the quenching of the fluorescence signal [24,25]. In order to solve the problem of dissolved oxygen in the system, nitrogen can be introduced into MSNs@R6G-PDDA/BSA-AuNCs.

Thermal stability determines whether a ratiometric fluorescent probe can be used for real-world testing. I_620_/I_553_ was obtained by heating MSNs@R6G-PDDA/BSA-AuNCs in water baths at different temperatures for 10 min and then performing fluorescence determination. As shown in Figure S3C, there is no significant change in I_620_/I_553_ at 4~50 °C, but I_620_/I_553_ begins to decrease gradually when the temperature exceeds 70 °C. This is because high temperatures alter the structure of BSA, which exposes AuNCs and causes fluorescence quenching. The fluorescent probes used in this work are stored in the dark at 4 °C, and the performance of the fluorescent probes will not change significantly. In practical applications, high-temperature environments are rare, so the fluorescent probes developed in this work have good application prospects.

In order to explore the effect of pH on fluorescent probes, PBS buffer solutions of different pH were added to MSNs@R6G-PDDA/BSA-AuNCs and fluorometric measurements were performed to obtain I_620_/I_553_. The results are shown in Figure S3D, where the values of I_620_/I_553_ are smaller under acidic conditions. With the increase of pH, the solution changes from acidic to alkaline, and the value of I_620_/I_553_ shows an upward trend. This is due to the presence of a large amount of -SH in BSA, and the alkaline environment enhances the reducing property of -SH, which, in turn, enhances the fluorescence performance of AuNCs [23]. The pH of fresh grass carp is about 7, and, with the increase of storage time, the pH of grass carp is between 6.5~7 [26]. Therefore, in practical applications, the fluorescence performance of fluorescent probes will not be greatly affected by the pH of grass carp samples.

Combined with the above experimental results, it can be seen that the ratiometric fluorescent probe developed in this work has good stability and can be applied to the detection of actual samples in complex environments.

### 2.5. Detection of H_2_O_2_

H_2_O_2_ can induce the oxidation of AuNCs leading to a fluorescence burst; based on this principle, BSA-AuNCs can be applied to the detection of H_2_O_2_ [27]. Various performance indicators of ratiometric fluorescent probes have been explored, as well as characterized previously, and the next step is to investigate the detection performance of H_2_O_2_. First, the effect of time response on the H_2_O_2_-induced fluorescence burst of MSNs@R6G-PDDA/BSA-AuNCs was investigated. Equal volumes of ultrapure water and 10 mM H_2_O_2_ were added to the system to mix and sonicate the reaction, and the fluorescence intensity was measured after different times of the reaction, respectively, to obtain I_620_/I_553_. The results are shown in Figure S4A. When H_2_O_2_ was not present in the system, the value of I_620_/I_553_ did not undergo any significant change. When H_2_O_2_ was added to the system, the value of I_620_/I_553_ decreased rapidly within 1 min, which was due to the oxidation of AuNCs induced by H_2_O_2_, which destroyed their structure and led to the fluorescence burst. The decrease in the value of I_620_/I_553_ slowed down after 3 min, and, with the prolongation of the time, the value of I_620_/I_553_ was no longer changed obviously, which was due to the interaction of H_2_O_2_ with AuNCs and the oxidization of AuNCs. H_2_O_2_ has reacted completely with AuNCs and the fluorescence of AuNCs is not changing. The above results indicate that the fluorescent probe has a fast and sensitive response to H_2_O_2_. In order to achieve the purpose of rapid detection, 3 min was finally chosen as the optimal response time of the probe to H_2_O_2_.

Based on the optimal response time, different concentrations of H_2_O_2_ were added to the system and the fluorescence intensity was detected to obtain I_620_/I_553_, respectively. The results are shown in Figure S4B. With the increase in H_2_O_2_ concentration, the value of I_620_/I_553_ decreased gradually. There was a good linear relationship between the H_2_O_2_ concentration and I_620_/I_553_ in the range of 0.5~1000 μM, and the linear regression equation was y = −1.22 × 10^−4^x + 0.88 (R^2^ = 0.998, and x is the H_2_O_2_ concentration). And the LOD value of this method was 0.5 μM. Therefore, this ratio sensing can be used for the quantitative detection of H_2_O_2_.

### 2.6. Discussion of DAO/BAs System

BAs can generate H_2_O_2_ catalyzed by DAO, while the ratiometric fluorescent probe has the potential to detect BAs indirectly because of the rapid and sensitive detection of H_2_O_2_ and the good linear relationship between H_2_O_2_ concentration and I_620_/I_553_. The incubation time, incubation temperature, DAO concentration, and pH were optimized for better sensitivity of the assay. Theoretically, equal amounts of cadaverine (Cad), histamine (His), and putrescine (Put) produced the same amount of H_2_O_2_ under the same conditions, which, in turn, had the same bursting effect on the ratiometric fluorescent probe. Therefore, Cad was chosen as a representative of BAs for the optimization of experimental conditions.

It takes time for DAO to catalyze the generation of H_2_O_2_ from BAs, so the effect of incubation time on the detection system needs to be explored. The results are shown in Figure S5A. The value of I_620_/I_553_ decreased from 20 to 30 min and no longer changed significantly after 30 min, which indicated that the enzymatic reaction was basically completed. In order to make the detection results more accurate and reliable, 30 min was chosen as the optimal incubation time to fully decompose the BAs. The results of the incubation temperature optimization are presented in Figure S5B. The ratio of I_620_/I_553_ reached its lowest value at 37 °C. Considering the associated error, this may be due to the enzyme reaction being most efficient around 37 °C. Thus, 37 °C was determined to be the relatively optimal temperature for subsequent experiments. The concentration of DAO also affects the detection system; for this reason, different concentrations of DAO were added separately for the experiments. The results are shown in Figure S5C. The values of I_620_/I_553_ show a tendency to decrease and then increase as the concentration of DAO increases. This is because, at a DAO concentration of 30 mg/mL, the DAO-catalyzed hydrolysis of BA produces H_2_O_2_, which can effectively participate in the oxidation of BSA-AuNCs. However, when the DAO concentration exceeds 30 mg/mL, the hydrolysis reaction rate increases, leading to a higher concentration of H_2_O_2_ in the system. As a result, some H_2_O_2_ may escape before it has the opportunity to participate in the oxidation of BSA-AuNCs. The concentration of DAO at 30 mg/mL was finally selected for subsequent experiments to obtain a more sensitive detection system. pH also has an effect on the catalytic efficiency of DAO, so experiments were conducted using different pH environments. The results are shown in Figure S5D. The value of I_620_/I_553_ is the smallest when the pH is 7.2, which indicates that the fluorescence burst effect is the best at this time, which is due to the best catalytic effect of DAO at pH 7.2, which was finally selected as the optimum pH.

### 2.7. Detection of BAs

**Figure 5:**
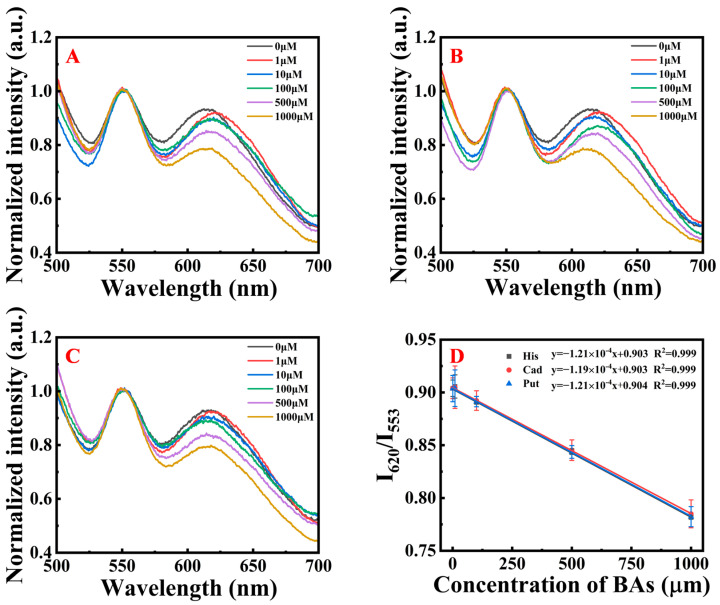
The fluorescence spectra of MSNs@R6G-PDDA/BSA-AuNCs with addition of different concentrations of BAs: (**A**) His; (**B**) Cad; and (**C**) Put. (**D**) The standard curve between the fluorescence intensity ratio I_620_/I_553_ of MSNs@R6G-PDDA/BSA-AuNCs and BAs (His, Cad, and Put) concentrations.

On the basis of the above optimal conditions, the quantitative detection of BAs (Cad, His, and Put) was explored. Different concentrations of BAs were added to the detection system and fluorescence detection was performed to obtain fluorescence profiles, respectively. The results are shown in Figure 5A–C. As the concentration of BAs increases, the fluorescence burst occurs, so that the value of I_620_/I_553_ shows a decreasing trend. As shown in Figure 5D, the fluorescence intensity ratio of the ratiometric fluorescent probe, I_620_/I_553_, was closely correlated with the BAs concentration and had a good linear relationship in the range of 0 to 1000 μM. The linear regression equations for BAs were Cad: y = −1.19 × 10^−4^x + 0.903 (R^2^ = 0.999); Put: y = −1.21 × 10^−4^x + 0.904 (R^2^ = 0.999); and His: y = −1.21 × 10^−4^x + 0.903 (R^2^ = 0.999), where y is the value of I_620_/I_553_ and x is the concentration of BAs. The LOD for BAs is 0.1 μM (S/N = 3). Meanwhile, by observing that the three linear regression curves almost overlapped, which indicated that the ratiometric fluorescent probe had the same bursting effect on three different BAs.

As shown in Table S1, comparing the assay developed in this work with the past assays for BAs, both the detection range and the LOD were more superior, which indicates that the ratiometric fluorescent probe developed in this work has excellent detection performance. In addition to this, ratiometric fluorescent probes are able to avoid interference from background fluorescence.

### 2.8. Specificity and Reproducibility of Ratiometric Fluorescent Probe

There are many other substances present in the actual sample assay, which can affect the detection of the target. Therefore, the ratio fluorescent probe needs to have good anti-interference ability. In order to test the specificity of the ratiometric fluorescent probe for the detection of BAs, amino acids (Hist, Tyr, Lys, and Phe) and metal ions (NaCl, KCl, CaCl_2_, and MgSO_4_) were used as interferences. As shown in Figure S6A, only His, Cad, and Put caused fluorescence quenching, which, in turn, led to a decrease in the values of I_620_/I_553_. There was no significant change in I_620_/I_553_ with the addition of other interferences compared with the blank group, indicating that the ratio fluorescent probe was able to specifically detect BAs (His, Cad, and Put).

In practice, ratiometric fluorescent probes can be affected by shelf life, preparation batch, and so on. In order to investigate the performance of the ratiometric fluorescent probe, the repeatability, reproducibility, and stability of the probe were tested. BAs were added to different batches and the same batch of MSNs@R6G-PDDA/BSA-AuNCs for the fluorescence assay, respectively, in parallel three times. As shown in Figure S6B,C, there was no significant difference in the values of I_620_/I_553_, and the RSD values are 0.32% and 1.65% (less than 5%), respectively, indicating that the method has good accuracy. In addition, to test the stability of the fluorescent probes at this ratio, the same concentration of BAs was detected using the same lot of MSNs@R6G-PDDA/BSA-AuNCs on Day 1 and Day 30. As shown in Figure S6D, the difference between I_620_/I_553_ was less than 5%, indicating that the ratio fluorescent probe has good stability. Based on the above results, the ratio fluorescent probe has a good prospect in the detection of BAs.

### 2.9. Detection of Real Sample

In order to further evaluate the practicability of the ratiometric fluorescent probe developed in this work in the detection of actual samples, grass carp samples were quantitatively spiked with BAs to determine the recovery. As shown in Table 1, the recovery rate of BAs was 97.57~104.29%, and the RSD value was 0.1~1.1% (less than 5%), which indicated that the method was used for the detection of BAs in actual grass carp samples. The t_exp_ of 0.89 is well below the cut-off value of 2.57, confirming that the probe’s accuracy falls within the 95% confidence level. The hypothesis test further verified the precision of the ratio fluorescent probe for the detection of BAs.

In addition, a 10-fold diluted extract (PBS, 0.2 M, pH = 7) from the edible portion of fresh grass carp was used as a matrix for detection, where the influence of the original BAs in the matrix could be considered negligible. Based on the linear range shown in Figure 5, the signal ratio (I_620_/I_553_) of MSNs@R6G-PDDA/BSA-AuNCs exhibits a linear relationship with the concentration of BAs (Figure S7). The detection limit in real grass carp samples was 0.17 μM, indicating the suitability of this probe for detecting BAs in practical applications.

## 3. Materials and Methods

### 3.1. Materials and Chemicals

Unless other stated, all chemicals and solvents are at least analytical grade. Poly diallyldimethylammonium chloride (PDDA) was bought from Aladdin Bio-Chem Technology Co., Ltd. (Shanghai, China). Rhodamine 6G (R6G), diamine oxidase (DAO), and histamine (His) were received from Sigma-Aldrich (Shanghai, China).

The fresh grass carp samples were purchased from a local supermarket in Jingzhou (Hubei, China) for subsequent actual samples’ detection.

### 3.2. Characterization and Instrumentation

The fluorescence spectrum, ultraviolet-visible light (UV–Vis) absorption spectrum and Fourier-transform infrared (FT-IR) spectrum were obtained by F-7000 fluorescence spectrophotometer (Hitachi Limited, Tokyo, Japan), TU-1900 double beam UV–Vis spectrophotometer (Beijing Purkinje GENERAL Instrument Co., Ltd, Beijing, China) and IR-960 FT-IR spectrophotometer (Tianjin Ruian Technology Co., Ltd, Tianjin, China), respectively. The transmission electron microscope (TEM) measurements were conducted on HT-7800 transmission electron microscope (Hitachi Limited, Tokyo, Japan). The zeta potential and particle size were performed on Zeta-sizer Nano ZS90 dynamic light scatterer (Malvern Panalytical Ltd., Malvern, UK).

### 3.3. Synthesis of MSNs@R6G-PDDA/BSA-AuNCs

BSA-AuNCs [28] with MSNs@R6G [29,30,31] were synthesized according to previous literature. Specific synthesis methods are in the Supplementary Materials.

The prepared MSNs@R6G stock solution was diluted 15-fold to obtain MSNs@R6G solution with a concentration of 0.33 mg/mL. Take 30 mL of the above solution and add it to the reaction flask under light-avoidance conditions with medium speed stirring; then, add 3 mL of 20% poly-diallyldimethylammonium chloride (PDDA) and stir for 2 h under light-avoidance conditions, to obtain 14.85 mg/mL of MSNs@R6G-PPDA solution. Take 20 mL of MSNs@R6G-PPDA solution and 20 mL of BSA-AuNCs solution in a reaction vial and mix them well; then, place the reaction vial in a water bath at 30 °C for 20 min at 1300 rpm/min. Finally, 8.5 mg/mL of MSNs@R6G-PDDA/BSA-AuNCs ratiometric fluorescent probe was obtained.

### 3.4. Detection Methods of BAs

Configure standard solutions of BAs (His, Put, and Cad) at concentrations of 0.5, 5, 10, 50, 100, 500, and 1000 μM. Take 20 μL of DAO (30 mg/mL, enzyme activity ≥ 0.05 units/mg) with pH = 7.2 and mix it with 180 μL of BAs standard solution with pH = 7 and incubate at 37 °C for 30 min. Under the condition of room temperature, the above mixture was mixed well with 200 μL MSNs@R6G-PPDA/BSA-AuNCs solution, and the fluorescence signals of the above mixture were measured and recorded after sonication reaction for 3 min. The measured parameters were λex 370 nm, slit width of 10 nm, and wavelength range of 500~700 nm.

### 3.5. Monitoring BAs in Actual Samples

The actual sample selected was grass carp. BAs in fish samples were collected based on previous literature with slight modifications [32]. A 10 g sample of grass carp was mixed with 20 mL of 10% TCA and homogenized at high speed for 5 min. The resulting mixture was centrifuged at 1000 rpm/min for 10 min and the supernatant was taken. Then, 20 mL of hexane was added to the supernatant and vortexed for 5 min with the aim of removing fat. After standing and layering, the upper organic phase was removed and 10 mL of the lower supernatant was collected. The supernatant was diluted to a suitable concentration using 0.2 mol/L pH = 7 phosphate buffer solution. Different concentrations of BA standards were added to the actual samples, and 180 μL of the above BA mixture was added with 20 μL of 30 mg/mL DAO (pH = 7.2) and incubated at 37 °C for 30 min; then, an equal volume of ratiometric fluorescent probe solution was added to the above mixture and ultrasonic reaction was carried out for 3 min under the condition of room temperature, and the ratiometric fluorescent signals were recorded.

## 4. Conclusions

In summary, a ratiometric fluorescent probe based on MSNs@R6G-PDDA/BSA-AuNCs was developed for the detection of BAs. The H_2_O_2_ produced by the hydrolysis of BAs catalyzed by DAO oxidizes AuNCs, resulting in the red fluorescence quenching of AuNCs. The yellow fluorescence of R6G is not affected, and a self-calibration system is formed with BSA-AuNCs, which improves the anti-interference ability of the probe. The study of BAs/DAO and the optimization of the preparation conditions achieved a linear detection of BAs in the range of 1–1000 μM with a LOD value of 0.1 μM. The cascade reaction of DAO makes this strategy have good specificity and stability for the detection of BAs. In the detection of actual grass carp samples, the recovery rate of spiked grass carp was 97.57~104.29%, and the RSD was 0.1~1.1%, which proved that the strategy was reliable in practical application.

## Figures and Tables

**Table 1:** Recoveries of BA detection in grass carp samples and statistical test.

Sample	Added (μM)				Found (μM)	Recovery (%)	RSD (%, *n* = 3)
	Cad	Put	His	Total			
**1**	**0**	**0**	0	0	165.29	/	/
2	20	0	0	20	190.08	102.59	0.1
3	0	30	0	30	195.59	100.15	1.1
4	0	0	120	120	297.52	104.29	0.2
5	60	60	60	180	355.37	102.92	0.4
6	30	60	120	210	369.15	98.36	0.2
7	150	60	20	230	385.67	97.57	0.2
Recovery analysis							
Mean recovery R_exp_					100.98		
Standard deviation of recoveries (S_R_)					2.70		
Number of test samples (N)					6		
t_exp_					0.89		
t(0.05,5)					2.57		

## Data Availability

Data are contained within the article or Supplementary Materials.

## Supplementary Materials

The following supporting information can be downloaded at: https://www.mdpi.com/article/10.3390/ijms26010139/s1. References [33,34,35,36,37,38,39,40] are cited in the supplementary materials.
