# Supplementary material for: Development of Sensitive R6G/AuNCs Ratiometric Fluorescent Probes for the Detection of Biogenic Amines in Fish Products
Source: Int J Mol Sci. 2024 Dec 27;26(1):139. doi: 10.3390/ijms26010139 (PMC11720474; doi:10.3390/ijms26010139)
Supplement: Supplementary file 1 [file ijms-26-00139-s001.zip › ijms-3314082-supplementary.pdf]

Supplementary

Development of Sensitive R6G/AuNCs Ratiometric Fluorescent Probes for the Detection of Biogenic Amines in Fish Products

Yutong Huang[†,1], *Simiao Zhang* [†,1], *Mei zhou*[1], *Xiaokang Xu*[1], *Weiqing Sun*[1], *Jing Ma*[*,1], *Long Wu*[*,]

[1]College of Life Science, Yangtze University, Jingzhou 434023, China.

[2]School of Food Science and Engineering, Key Laboratory of Tropical and Vegetables Quality and Safety for State Market Regulation, Hainan University, Haikou 570228, China.

Table of contents

Synthesis of BSA-AuNCs

Synthesis of MSNs@R6G

Fig. S1. TEM of MSNs

Fig. S2. Excitation emission fluorescence matrix of (A) MSNs@R6G; (B) BSA-AuNCs; (C) MSNs@R6G-PDDA/BSA-AuNCs, inset：the photographs under ultraviolet light.

Fig. S3. Stability evaluation of the probe (A) photostability; (B) storage stability; (C) thermostability; (D) effect of pH on fluorescence stability of the probe.

Fig. S4. Time response of MSNs@R6G-PDDA/BSA-AuNCs with and without H_2O_2； (B) The fluorescence intensity ratio I_{620}/I_{553} of MSNs@R6G-PDDA/BSA-AuNCs as a function of H_2O_2 concentration

Fig. S5. (A) Option of the optimum incubation time; (B) Option of the optimum incubation temperature; (C) Option of the optimum concentration of DAO; (D) Option of the optimum pH of DAO.

Fig. S6. (A) Selectivity; (B) repeatability; (C) reproducibility; (D) stability of MSNs@R6G-PDDA/BSA-AuNCs for BAs detection.

Fig. S7. The fluorescence spectra of MSNs@R6G-PDDA/BSA-AuNCs with addition of different concentrations of BAs: (A) Cad; (B) His; (C) Put. (D)The standard curve between the fluorescence intensity ratio I_{620}/I_{553} of MSNs@R6G-PDDA/BSA-AuNCs and BAs (Cad, His, Put) concentrations.

Table S1. Performance comparison of different methods or BAs detection.

Synthesis of BSA-AuNCs

To prepare BSA-AuNCs, 5 mL of 10 mM $HAuCl_4$ solution and 5 mL of 50 mg/mL BSA solution were mixed and stirred vigorously at 37°C for 5 min to ensure homogeneity. Subsequently, 1 M NaOH was added to adjust the pH of the reaction mixture to 12. The mixture was then incubated at 37°C under light-protected conditions, stirring at 780 rpm for 12 h. During this process, the solution's color changed from yellow to a uniform reddish-brown, indicating the formation of BSA-AuNCs.

The resulting BSA-AuNCs stock solution was transferred to an ultrafiltration centrifuge tube with a molecular weight cut-off of 30 kDa. Ultrapure water was added, and the solution was filtered and centrifuged at 2500 rpm for 10 min. This process was repeated four times to remove unreacted substances. After discarding the filtrate, ultrapure water was added to restore the solution to its original volume(25.3 mg/ mL).

The purified BSA-AuNCs solution was diluted 5-fold (5.06 mg/ mL) and stored at 4°C.

Synthesis of MSNs@R6G

A mixture of 20 mL of R6G solution (0.02 mg/mL) and 100 mg of MSNs powder was stirred at medium speed at room temperature under light-protected conditions for 2 h. Upon completion of the reaction, the unincorporated R6G was removed by washing the mixture twice with ultrapure water, followed by centrifugation at 10,000 rpm for 10 minutes. The resulting precipitate, MSNs@R6G, was dispersed in 20 mL of ultrapure water for further use. The concentration of MSNs@R6G in the final solution was 4.9 mg/mL.

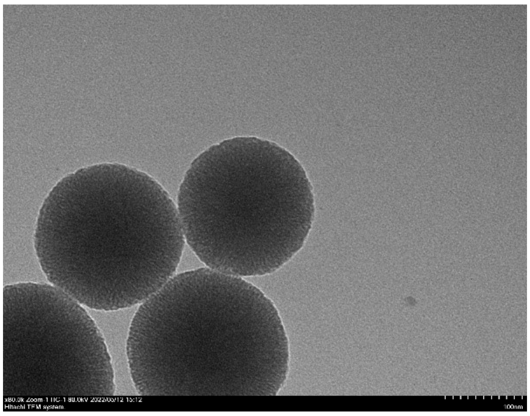

Fig.S1. TEM of MSNs

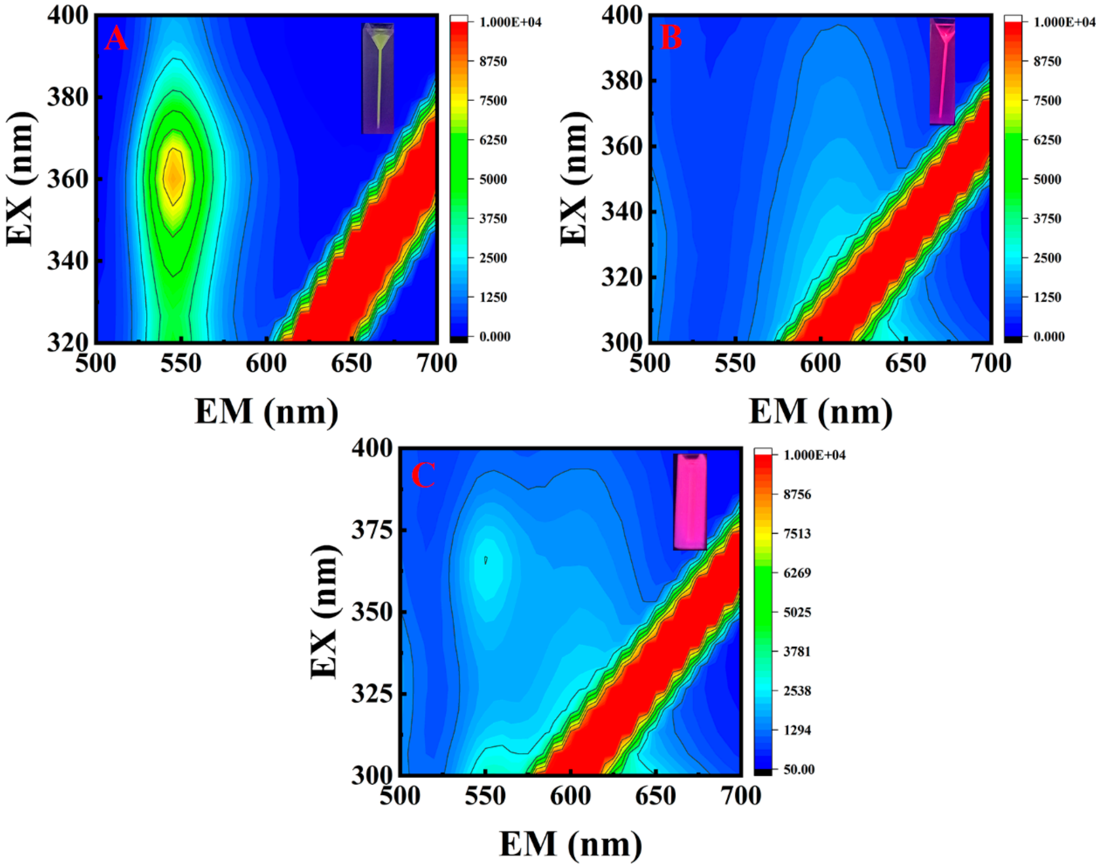

Fig. S2. Excitation emission fluorescence matrix of (A) MSNs@R6G; (B) BSA-AuNCs; and (C) MSNs@R6G-PDDA/BSA-AuNCs, inset：the photographs under ultraviolet light.

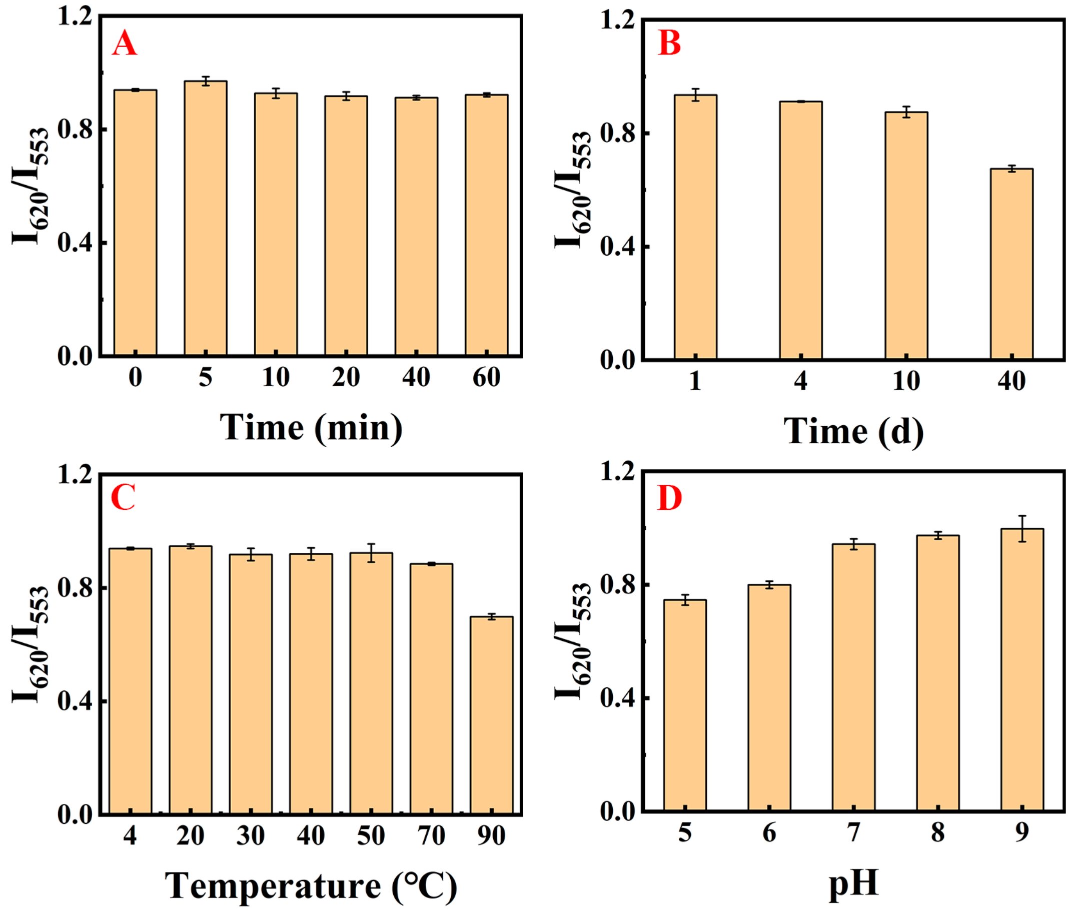

Fig. S3. Stability evaluation of the probe (A) photostability; (B) storage stability; (C) thermostability; and (D) effect of pH on fluorescence stability of the probe.

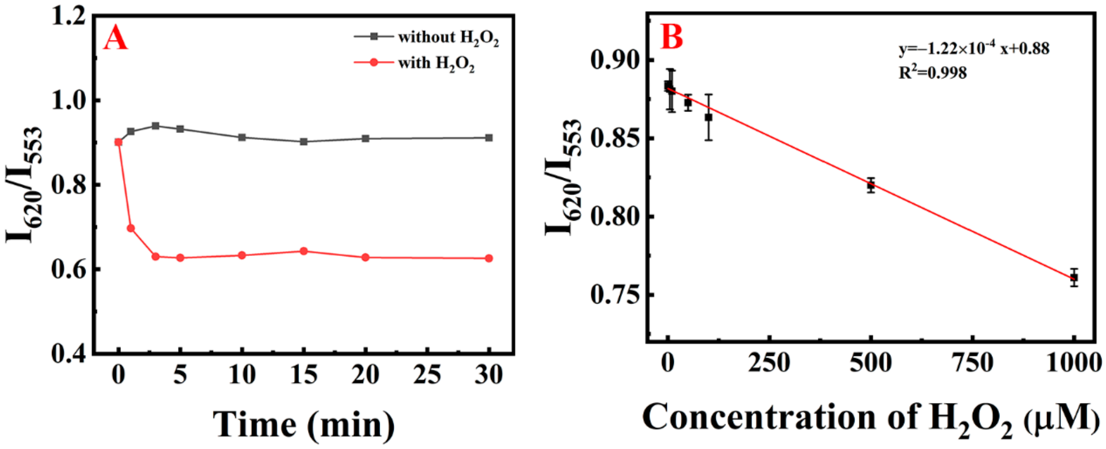

Fig. S4. (A)Time response of MSNs@R6G-PDDA/BSA-AuNCs with and without H_2O_2; (B) The fluorescence intensity ratio I_{620}/I_{553} of MSNs@R6G-PDDA/BSA-AuNCs as a function of H_2O_2 concentration.

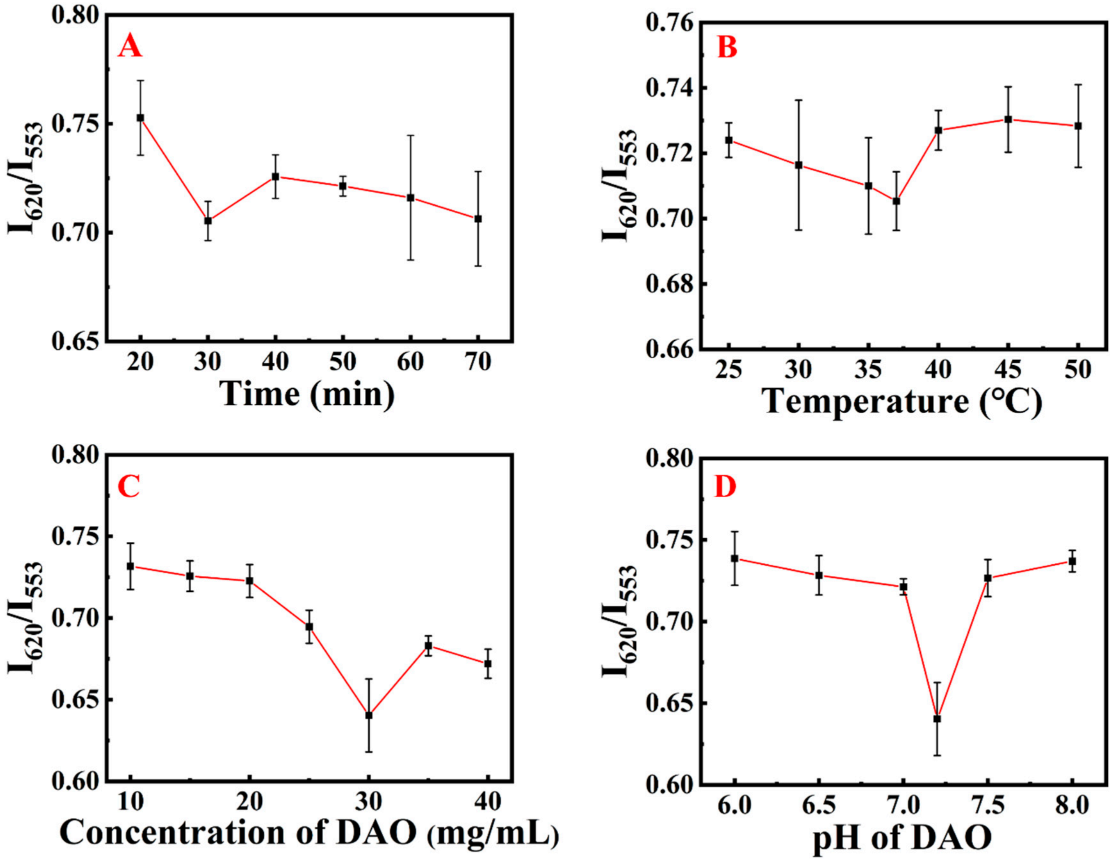

Fig. S5. (A) Option of the optimum incubation time; (B) option of the optimum incubation temperature; (C) option of the optimum concentration of DAO; (D) option of the optimum pH of DAO.

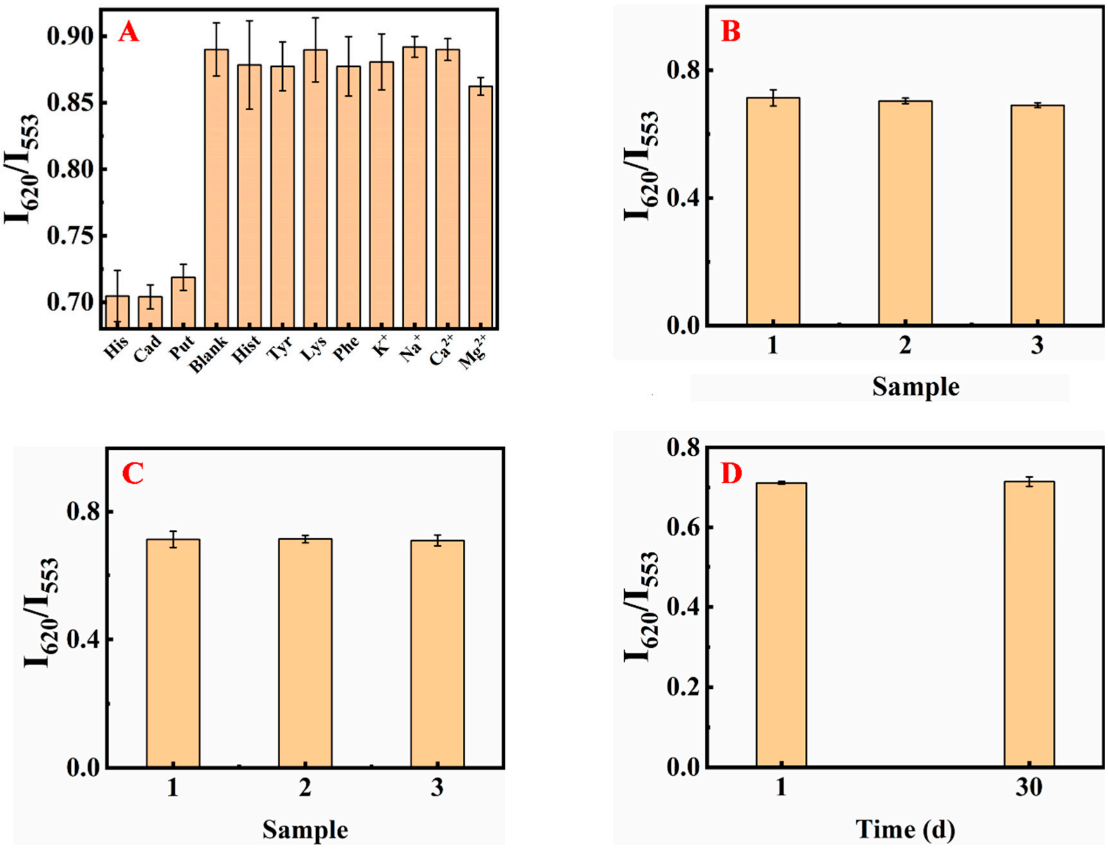

Fig. S6. (A) Selectivity(His, Cad, and Put: 1 mM; others: 10 mM); (B) repeatability (Samples 1, 2, and 3 represent parallel test groups within the same batch); (C) reproducibility (Samples 1, 2, and 3 represent samples from different preparation batches); (D) stability of MSNs@R6G-PDDA/BSA-AuNCs for BAs detection.

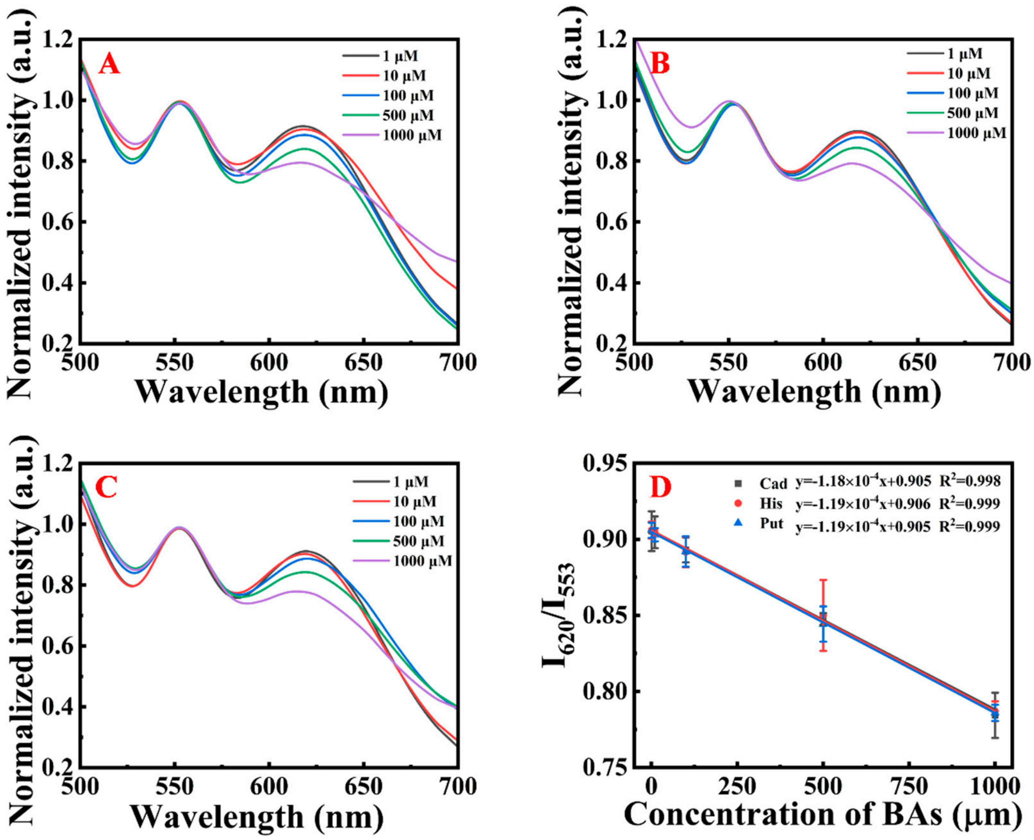

Fig. S7. The fluorescence spectra of MSNs@R6G-PDDA/BSA-AuNCs with addition of different concentrations of BAs: (A) Cad; (B) His; and (C) Put. (D)The standard curve between the fluorescence intensity ratio I_{620}/I_{553} of MSNs@R6G-PDDA/BSA-AuNCs and BAs (Cad, His, and Put) concentrations.

Table S1. Performance Comparison of Detection Methods for H_2O_2 and BAs

Probes	linear range (μM)	LOD (μM)	Ref.
CS-graft-OA micelles	0~50	10	[1]
Eu/Tb(HFA)@clay	35.6~197.9	1.23	[2]
TGA-CdTe QDs	9.6~570	9.6	[3]
N, S-CDs@CdTe QDs	0~50	3.886	[4]
ZnO@PLP QDs	2.49~24.4	0.59	[5]
BCy-Biotin	0~80	0.182	[6]
Cellulose nanofibrils/carbon dots composite	6~42	0.93	[7]
Au@Ag NRs Au@Ag NR	10~10000	6	[8]
MSNs@R6G-PDDA/BSA-AuNCs	1~1000	0.1	this work

References

1. Zhong, H.Q.; Liu, C.C.; Ge, W.J.; Sun, R.C.; Huang, F.; Wang, X.H. Self-Assembled Conjugated Polymer/Chitosan-graft-Oleic Acid Micelles for Fast Visible Detection of Aliphatic Biogenic Amines by "Turn-On" FRET. *ACS Appl. Mater. Interfaces* **2017**, *9*, 22875–22884.
2. Guo, L.; Tian, X.; Zhu, C.; Hussain, S.; Han, J.; Li, H., A dual-emission fluorescent ratiometric probe based on bimetallic lanthanide complex interacted in nanoclay for monitoring of food spoilage. *Sens. Actuators B Chem.* **2022,** 366.
3. Khan, S.; Carneiro, L.S. A.; Vianna, M.S.; Romani, E.C.; Aucelio, R.Q., Determination of histamine in tuna fish by photoluminescence sensing using thioglycolic acid modified CdTe quantum dots and cationic solid phase extraction. *JOURNAL OF LUMINESCENCE* **2017**, *182*, 71–78.
4. Yan, J.; Fu, Q.; Zhang, S.; Liu, Y.; Shi, X.; Hou, J.; Duan, J.; Ai, S., A sensitive ratiometric fluorescent sensor based on carbon dots and CdTe quantum dots for visual detection of biogenic amines in food samples. *Spectrochim. Acta A Mol. Biomol. Spectrosc.* **2022,** 282.
5. Yadav, A.; Upadhyay, Y.; Bera, R.K.; Sahoo, S.K., Vitamin B6 cofactors guided highly selective fluorescent turn-on sensing of histamine using beta-cyclodextrin stabilized ZnO quantum dots. *Food Chem.* **2020,** 320.
6. Zhong, D.; Xiong, S.; Zhang, Y.; Cui, M.; Liu, L.; Xu, Y.; Wang, P.; Zhang, W., H2O2-activated NIR fluorescent probe with tumor targeting for cell imaging and fluorescent-guided surgery. *Sens. Actuators B Chem.* **2024**, *418*, 136249.
7. Bandi, R.; Alle, M.; Park, C.-W.; Han, S.-Y.; Kwon, G.-J.; Kim, N.-H.; Kim, J.-C.; Lee, S.-H., Cellulose nanofibrils/carbon dots composite nanopapers for the smartphone-based colorimetric detection of hydrogen peroxide and glucose. *Sens. Actuators B Chem.* **2021**, *330*, 129330.
8. Han, L.; Li, C.; Zhang, T.; Lang, Q.; Liu, A., Au@Ag Heterogeneous Nanorods as Nanozyme Interfaces with Peroxidase-Like Activity and Their Application for One-Pot Analysis of Glucose at Nearly Neutral pH. *ACS Appl. Mater. Interfaces* **2015**, *7*, 14463–14470.
